# Design and Analysis of a Hybrid-Type RF MEMS Phase Detector in X-Band

**DOI:** 10.3390/mi13050786

**Published:** 2022-05-18

**Authors:** Juzheng Han, Dazhi Ding

**Affiliations:** Department of Communication Engineering, Nanjing University of Science and Technology, Nanjing 210094, China; dzding@njust.edu.cn

**Keywords:** microwave wave detection, micro-electromechanical systems (MEMS), phase shift measurement, thermoelectric sensor

## Abstract

In this paper, we have designed, analyzed, and characterized a hybrid-type MEMS device for X-band phase shift measurement. The signal related to a phase shift of the inputs is fractionally in-line coupled by a MEMS beam and delivered to a thermoelectric power sensor, where the phase is ultimately converted into DC voltage output. With the hybrid of the MEMS beam and the thermoelectric power sensor, both in-line detection process and phase-DC voltage conversion is reserved, which is a benefit for large power capacity, good linearity property, and high-level integration density. In order to get a deep insight into the physical mechanisms involved in the phase detection process, a comprehensive analysis model is presented. The beam is modeled as a precise RLC circuit component, where the capacitance is related to the input power. The fabrication is compatible with GaAs monolithic microwave integrated circuit (MMIC) technology. Experimental results show that return loss is smaller than −11.3 dB and isolation is better than −9.3 dB over X-band. Phase shift detection from 0 to 180 degrees is verified for a large power range of 200–1600 mW (23–32 dBm). The perfect linearity property of the phase-detection sensitivity is demonstrated in the same power range. Low intermodulation distortion is also confirmed through measurement. It is revealed from the comparison between this work and other published results in the literature that this presented hybrid-type structure shows superiorities in both power handling ability and phase-detection linearity. It can be adopted in medium power signal applications with a high level of integration.

## 1. Introduction

The characterization of phase angles of the microwave signal is at the base of many communication applications, such as frequency synthesizers, modulators, demodulators, and phased-array transceivers [1,2,3]. The basic implementation method for phase angle measurement is based on evaluating the relative phase shift between the test signal and the reference signal in two different channels and converts the phase information into other kinds of signals that are easy to detect and read out. To date, many designs for phase measurements have been proposed through solid-state circuits with diodes, multipliers, and mixers [4,5,6,7], and magnetic tunnel junctions [8,9], et al.

For the last few decades, radio frequency devices based on micro-electromechanical system (MEMS) technology, known as RF MEMS devices, have demonstrated significant preponderance compared to traditional solid-state devices [10]. Especially, compared to the above-mentioned counterparts, phase detectors based on MEMS technology feature many advantages in terms of low power consumption, small occupied size, and a high-quality factor. Distinguished by the signal conversion method and the output form, previous RF MEMS devices for phase measurement in literature can be classified into two categories. The first one is named the terminated-type phase detector established by a power combiner cascaded with a thermoelectric sensor [11,12]. The signal at the combined port which contains phase information would be directly absorbed by the thermopiles, partly converted into DC voltage while the rest dissipated in the substrate. This DC voltage can be fed directly to a voltage-controlled oscillator at a phase-locked loop platform, which helps to eliminate the use of a low pass filter, decrease the circuit complexity and improve the integration density of the system. However, these terminated-type phase detectors present drawbacks on linearity degradation, risk of over-burning, and limited power handling ability due to impressive temperature rise in the substrate under high input power [13]. For the second type, which operates in an in-line manner, a capacitive sensor based on a MEMS beam serves as a substitution for the thermoelectric sensor [14]. Different from the terminated-type phase detectors, where the input signal is totally consumed by the thermoelectric power sensor, the in-line manner means the device can couple a certain percentage of the transmitted microwave signal from the signal line for detection, while the rest of the microwave signal can be reused after detection or transmit to the back-end circuit. In the in-line type phase detector, the signal is fractionally coupled in an in-line manner by the capacitive beam, and the phase is eventually obtained by measuring the capacitance change caused by the displacement of the beam. This in-line method can expand the dynamic range and overcome the over-burning issue. Nevertheless, the linearity is still limited due to the nonlinear response of the beam under large input. Moreover, as the capacitance is internally inserted under the beam, it is more difficult to be integrated with other circuits. Thus, neither of the two kinds of RF MEMS phase detectors can satisfy special and synthetical application occasions, where in-line detection process, DC output, large power capacity and linear range, and high-level integration capability are required.

In order to seek a way to address the above issue, a new design of a hybrid-type RF MEMS phase detector is presented in this paper. The hybrid of a cantilever MEMS beam and a thermoelectric sensor is utilized and cascaded after a power combiner. The reference signal and the test signal with phase shift are injected into the detector through the power combiner. The cantilever MEMS beam functions to couple certain combined energy to the thermoelectric sensor, where the phase difference would be ultimately converted into DC voltage. Therefore, both the in-line detection procedure and DC voltage output are reserved. Moreover, as only fractional power is fed to the thermoelectric sensor, improved power handling ability and linearity property can be obtained. A comprehensive and precise analytical model is proposed to fully demonstrate the working principle of the presented structure. The cantilever beam is modeled as a RLC component, where the capacitance is related to the input power. Experiment results indicate that the phase detector can cover a bandwidth of 8–12 GHz (X-band) with return loss better than −11.3 dB and isolation better than −9.3 dB. The detection is capable of handling a phase range of 0–180° and a power range of 200–1600 mW (23–32 dBm). Detection sensitivity (DC output vs. phase shift) shows good linear and positive correlation with the input power. Both improved power capacity and enhanced linearity property are demonstrated compared with previously reported phase detectors. The application of this proposed structure lies in phase evaluation and calibration for medium power signal applications.

## 2. Design, Analysis, and Fabrication

### 2.1. Design and Analysis

**Figure 1:**
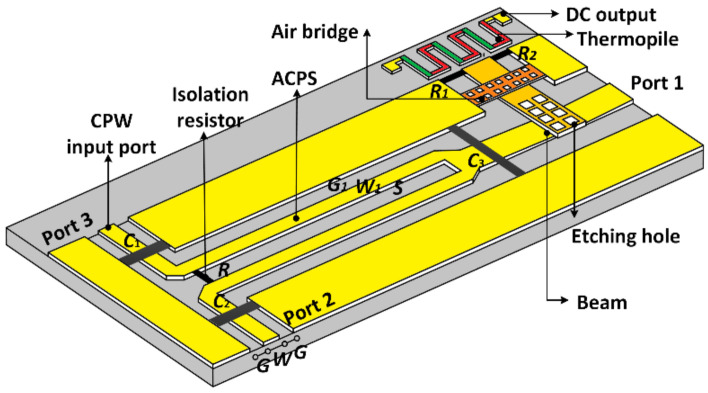
Schematic diagram of the proposed hybrid-type phase detector.

As depicted by the schematic diagram in Figure 1, the presented hybrid-type RF MEMS phase detector is architected in cascaded form by three parts: a power combiner, a cantilever MEMS beam suspended over the combined port of the power combiner, and a thermoelectric sensor cascaded at the fixed edge of the cantilever beam. In the power combiner, two channels (port 2 and port 3) with coplanar waveguides (CPWs) and asymmetrical coplanar strip lines (ACPSs) are provided for the test signal and the reference signal, resulting in a combined power related to the phase shift between them. Capacitors *C*_1_, *C*_2_, and *C*_3_ and resistor *R* inserted at the ACPSs function as efficient tools for size minimization and port isolation [15]. On the one hand, as the cantilever beam and the transmission line underneath can be regarded as a plate capacitor, part of the combined signal would be coupled by this capacitor and delivered to the thermoelectric sensor. The coupling capability is determined by the magnitude of the capacitance. On the other hand, the rest part of the combined signal would still transmit in an in-line way to the output port (port 1). Owing to this implementation, an in-line detection procedure is realized and the phase shift between the test and reference signal is eventually converted into a DC voltage output. Moreover, as only a fractional signal is utilized for phase characterization, the power at the thermoelectric sensor is much smaller than traditional terminated-type phase detectors. This means the presented phase detector can sustain a much higher power of the input. Thus, improved power handling ability and linearity property can be expected. Structure parameters of the presented hybrid-type phase detector are given in Table 1.

As mentioned above, the presented hybrid-type phase detector involves a series of physical mechanisms and signal conversion processes including signal transmission, capacitance coupling, and thermoelectric conversion. In order to fully explain the behavior of the presented phase detector, each part of the structure is investigated and analyzed in detail. The variables *V*_2_, *V*_3_ and *V*_c_ are the rms voltages of the test signal, the reference signal, and the combined signal, respectively. The relationship between them can be deduced by the vector combination principle as given in Equation (1).

(1) $$V_{c}=\sqrt{{\left(\frac{1}{\sqrt{2}}V_{2}\right)}^{2}+{\left(\frac{1}{\sqrt{2}}V_{3}\right)}^{2}+V_{2}V_{3}\cos\Delta \varphi }$$

The corresponding power of the test and the reference signals injected into port 2 and port 3 are characterized by magnitude *P*_2_ and *P*_3_, respectively. The combined power *P*_c_ obtained by the power combiner can be expressed by Equation (2).

(2) $$P_{c}=\frac{1}{2}\left(P_{2}+P_{3}\right)+\sqrt{P_{2}P_{3}}\cos\left(\Delta \varphi \right)$$

where Δ*φ* is the phase shift between the two inputs. The cosine relationship between phase shift Δ*φ* and combined power *P*_c_ is revealed, and the combined power reaches its maximum value when the two inputs are in-phase, while a minimum value can be observed for signals that are anti-phase. When the two input signals are characterized by the same magnitude *P*_in_, the expression in Equation (2) can be simplified as *P*_c_ = *P*_in_(1 + cos(Δ*φ*)).

According to the above analysis, when the combined power transfers through the transmission line beneath the cantilever beam, the power would be partly coupled out by the beam. The coupling capability relies on the capacitance between the beam and the signal line underneath. In Ref. [16], the coupling capacitance and coupling parameter are both regarded as a stable value, which couples a fixed percentage of the transmitted microwave power. As the beam would be actuated by the transmitted power and deviate from the initial position [10], the capacitance between the beam and the transmission line changes with the input power. Therefore, an increase in the input power will lead to an enhanced coupling capability.

**Figure 2:**
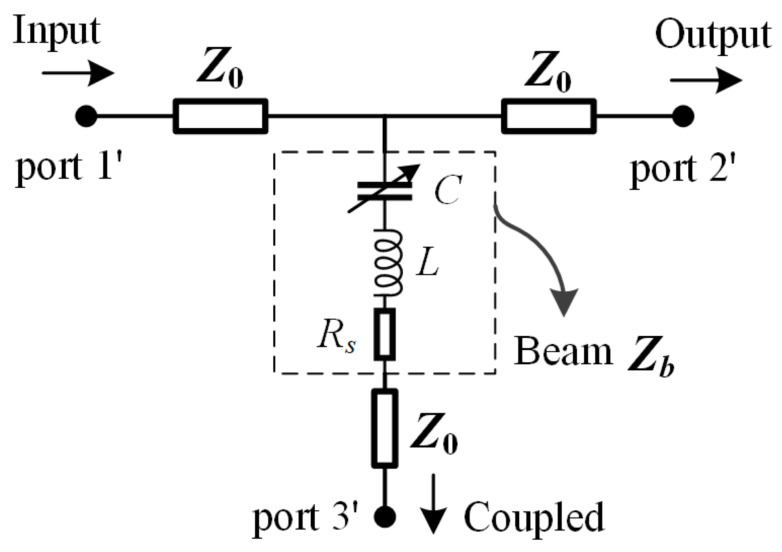
Equivalent circuit of the cantilever beam and the transmission line underneath.

In order to investigate the relationship between input power, deviation of the beam, and coupling capability of the beam, an equivalent circuit model of the beam and the transmission line underneath is established, depicted in Figure 2. It can be regarded as a three-port system, where port 1′ and port 2′ are the two ends of the transmission line, respectively, and port 3′ represents the cantilever beam for coupling. The superscript is utilized to distinguish from the port of the presented phase detector illustrated in Figure 1. The input signal at port 1′ is the combined power *P_c_* from the power combiner, fractional power is coupled by the beam and delivered to the thermoelectric sensor connected at port 3′, while the other part is transmitted in an in-line manner through port 2′. The beam can be equivalent to a RLC component, where *C* is the parallel plate capacitance, *L* is the inductance and *R_s_* is the series resistance of the beam. Expression for beam impendence *Z*_b_ is given in Equation (3).

(3) $$Z_{b}=\frac{1}{j\omega C}+j\omega L+R_{s}$$

For MEMS beams working in the microwave region, typical values for *R*_s_ are 0.1–0.2 Ω, and the inductance *L* is in the order of pH. The theoretical value for the inductance *L* is [17]

(4) $$L=2s[ln(\frac{2\pi s}{w})-1+\frac{w}{\pi s}]$$

where *s* is the distance between the edge of the signal line and the anchor of the beam.

The displacement of the beam is determined by the equilibrium state when the electrostatic force between the beam and the signal line equals the restoring force of the beam. The relationship governing the input power *P*_c_ and the beam deviation can be deduced by transmission line theory [18], as expressed by Equation (5).

(5) $$P_{c}=\frac{kx}{2\varepsilon_{0}AZ_{0}Z^{2}}{\left(g_{0}-x\right)}^{2}{\left(2Z_{b}+Z_{0}\right)}^{2}$$

where *k* is the elastic coefficient of the beam, *g*_0_ is the initial beam height, *x* is the beam deviation from the initial place, *A* is the overlap area of the beam and the transmission line underneath, *Z*_0_ is the characteristic impedance of the CPW which is 50 Ω in this design.

**Figure 3:**
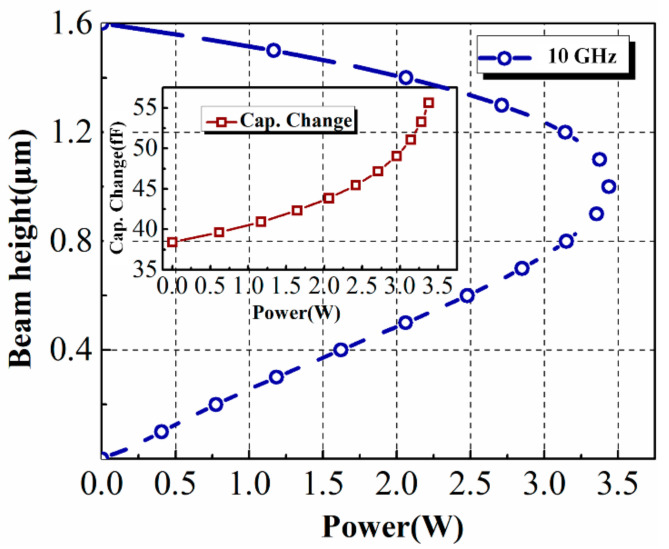
The calculated plot of the beam height under different power levels and the capacitance chance versus the input power.

Based on the mathematic analysis of Equation (5), the deviation and position of the beam can be obtained. Figure 3 gives the calculated plot of the beam height versus input power at 10 GHz. It is illustrated by the plot that “pull-in” happens at approximately 2/3 of the initial beam height, which is in accordance with the theory [10]. Moreover, linear and positive correlation between the beam deviation and the input power is revealed before the pull-in state. The variation of the parallel plate capacitance *C* under different power levels can also be obtained, presented in the insert of Figure 3. It is verified that the input power does have a non-negligible effect on the capacitance caused by the beam, especially under higher input.

Assuming that the displacement of the membrane flows in a one-dimensional model, the coupling capacitance can be expressed by

(6) $$C_{c}=C+C_{f}=\frac{\varepsilon_{0}A}{g_{0}-x}+C_{f}$$

where *C*_f_ represents the fringing capacitance which is about 20–60% of the parallel plate capacitance. Combining Equations (5) and (6), we can obtain the coupling capacitance under different input power levels.

According to the above analysis, the coupling capacitance increases with the input power, which would result in a variable coupling capability under different input power. To evaluate the coupled power by the coupling capacitance, an *S*-parameter model is needed for the three-port system in Figure 2. The return loss *S*_11_ at port 1′, insertion loss *S*_21_ between port 1′ and port 2′, and *S*_31_ representing the coupling capability of the beam can be deduced through transmission line theory, presented by Equations (7)–(9).

(7) $$S_{11}=-\frac{Z_{0}}{2Z_{b}+3Z_{0}}=-\frac{Z_{0}}{2\left(R_{s}+j\left(\omega L-\frac{1}{\omega C_{c}}\right)\right)+3Z_{0}}$$

(8) $$S_{21}=2\frac{Z_{b}+Z_{0}}{2Z_{b}+3Z_{0}}=2\frac{R_{s}+j\left(\omega L-\frac{1}{\omega C_{c}}\right)+Z_{0}}{2\left(R_{s}+j\left(\omega L-\frac{1}{\omega C_{c}}\right)\right)+3Z_{0}}$$

(9) $$S_{31}=\frac{2Z_{0}}{2Z_{b}+3Z_{0}}=\frac{2Z_{0}}{2\left(R_{s}+j\left(\omega L-\frac{1}{\omega C_{c}}\right)\right)+3Z_{0}}$$

**Figure 4:**
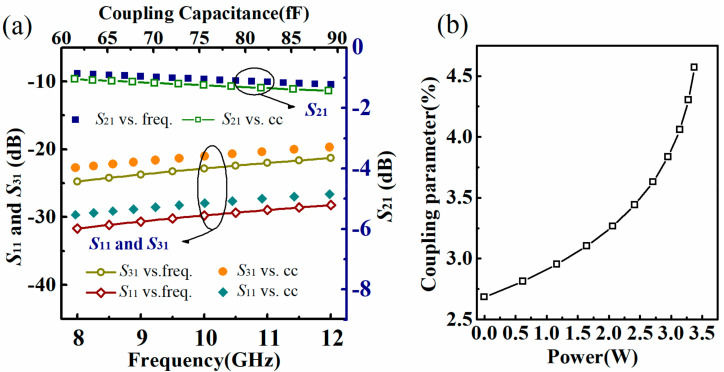
(**a**) Frequency spectrum of *S*-parameters and the relationship between *S*-parameters and coupling capacitance; (**b**) calculated plot of the coupling parameter versus input power.

The frequency spectrum of *S*-parameters (*S*-parameter vs. freq.) is given in Figure 4a for the initial beam state. Broadband property is demonstrated within X-band. Besides, the relationship between *S*-parameters and coupling capacitance (*S*-parameter vs. cc) is also given. It is shown from Figure 4a that the microwave performance deteriorates gradually both with the increase of the frequency and the coupling capacitance. This is primarily because of the dielectric loss in the substrate and the impedance mismatching caused by the deviation of the beam. By defining the coupling parameter *C*_p_ as the power ratio between the coupled power *P*_cp_ at port 3′ and the input power *P*_c_ in port 1′, it can be evaluated by *S*_31_ as

(10) $$C_{p}=\frac{P_{\mathrm{cp}}}{P_{c}}={\left|S_{31}\right|}^{2}$$

Combining Equations (5), (9) and (10), we can obtain the plot of the coupling parameter versus the input power, presented in Figure 4b.

The thermoelectric sensor which functions to convert the coupled power into a DC voltage output, is connected at the end of the beam through two load resistors and a section of transmission line. When the power coupled by the beam is introduced to the transmission line, it will be absorbed by the load resistors and converted into heat. As the heat conducts inside the thermoelectric sensor, a temperature gradient results along thermopiles. The two terminals of one thermocouple, distinguished by high and low temperatures, are generally called hot and cold junctions, respectively. According to the Seebeck effect, a DC voltage proportional to the temperature difference summation of all hot and cold junctions will be generated at the output pads. According to the above analysis, only a small portion of the input power is coupled by the beam and delivered to the load resistors, the thermoelectric power sensor can sustain a much higher input power level while still maintaining its linear detection behavior. Therefore, the power handling ability and linear property can be improved. The output DC voltage *V*_o_ can be expressed by the following equation.

(11) $$V_{o}=\left(\alpha_{1}-\alpha_{2}\right)\cdot P_{\mathrm{cp}}\cdot \overset{N}{\underset{i=1}{\sum }}\int_{r_{c}^{i}}^{r_{h}^{i}}\frac{1}{\lambda A\left(r\right)}dr=S\cdot P_{\mathrm{cp}}$$

where *α*_1_ and *α*_2_ are Seebeck coefficients of the two arms in the thermopiles, respectively, *P*_cp_ is the coupled power delivered to the load resistors which is converted to heat flow, *N* is the total number of the thermocouples, the integral term of 1/*λA*(*r*) represents the thermal resistance, and *S* is the sensitivity of the thermoelectric power sensor defined by output voltage versus input power.

**Figure 5:**
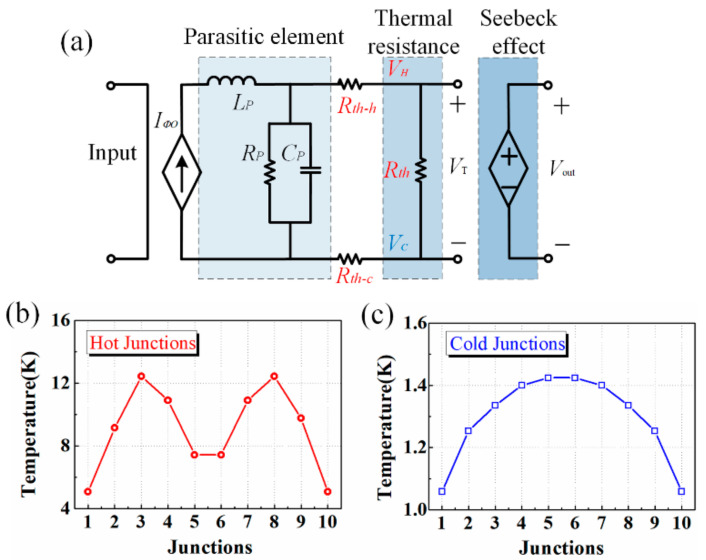
(**a**) Equivalent circuit model of the thermoelectric sensor, simulated temperature of (**b**) hot junctions and (**c**) cold junctions.

As illustrated in Figure 5a, an equivalent circuit model is also established for the thermoelectric sensor. According to analogies between thermal and electrical domains [19,20], heat flow and thermal resistance can be represented by electrical current and lumped resistor, respectively. As load resistors under microwave application are no longer pure resistors, parasitic elements should be taken into account and a certain amount of the input power will be stored inside them. Besides, the Seeback effect is modeled as a voltage-controlled voltage source. The temperature of hot, and cold junctions and the total temperature difference, symbolized by voltage in the circuit, can be predicted by computer-aided circuit simulators, such as Advanced Design System (ADS). For a thermoelectric sensor with 10 pairs of thermocouples, the simulated temperatures of the hot and cold junctions at 10 GHz, 200 mW are plotted in Figure 5b,c.

Based on the above analysis, the relationship between phase difference Δ*ϕ* and the output DC voltage *V*_o_ can be eventually demonstrated by the following formula.

(12) $$V_{o}=S{\left|S_{31}\right|}^{2}\left[\frac{1}{2}\left(P_{1}+P_{2}\right)+\sqrt{P_{1}P_{2}}\cos\left(\Delta \varphi \right)\right]$$

For both in-line type and terminated-type RF MEMS detectors, the phase detection sensitivity degrades with the increase of the input power. This is primarily because of the nonlinear response of the beam under high power level [21] and the thermal conductivity of the GaAs substrate decreases at high temperature. However, for the hybrid-type phase detector presented in this work, the cantilever beam, and the thermoelectric sensor function separately. The coupling capability increases with the input power, while the power fed to the thermoelectric sensor is much smaller than the terminated-type phase detector under the same input power. Therefore, a larger power capacity and enhanced linearity property can be expected.

### 2.2. Fabrication

**Figure 6:**
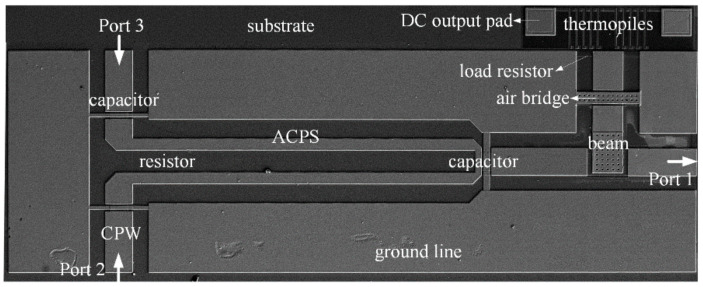
Scanning electron microscope (SEM) photograph of the proposed hybrid-type phase detector.

The proposed hybrid-type phase detector is fabricated using GaAs monolithic microwave integrated circuit (MMIC) process. SEM photograph of the fabricated hybrid-type MEMS phase detector is presented in Figure 6. The total occupied size is about 2.45 mm^2^. Manufacturing processes developed for this structure are described as follows.

(a) The transmission lines and the electrode pads are fabricated by evaporating an Au layer and an adhesive AuGeNi/Au layer. Then Ti/Au/Ti seed layer is evaporated and patterned. After that, the top Ti layer is removed.
(b) The two arms of the thermopiles are made of N+ GaAs and Au, respectively. Specifically, N+ GaAs is fabricated using the ion implantation method, and Au is sputtered with a lift-off process.
(c) Resistors are made by the TaN layer through depositing. The square resistance of the material is 25 Ω/square.
(d) In order to manufacture the cantilever MEMS beam, a sacrificial layer of polyimide is firstly patterned. Then Au is etched to form the beam. The sacrificial layer is removed after that.
(e) Finally, in order to reduce the power dissipated inside the substrate, the substrate underneath the load resistors and the thermopiles is back-etched through dry etching technology.

## 3. Experiments and Discussions 

To evaluate the overall property of the proposed hybrid-type phase detector, measurements of microwave performance and phase shift detection capability are conducted.

### 3.1. Microwave Performance 

**Figure 7:**
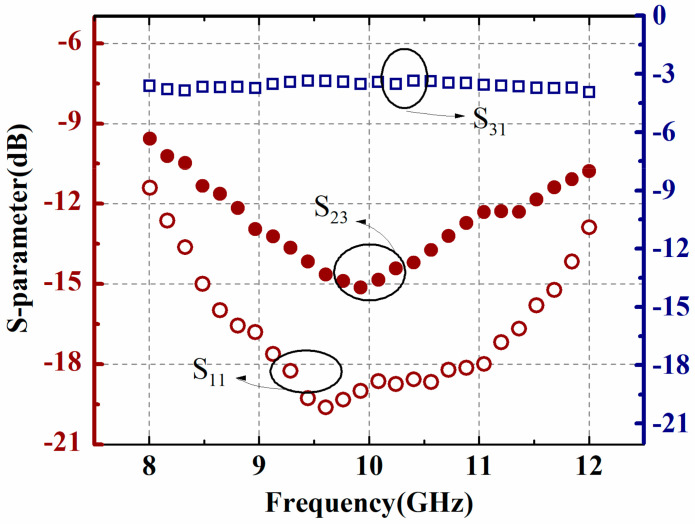
Measured results of *S*_11_, *S*_31,_ and *S*_23_.

*S*-parameters of the presented phase detector are measured by a vector network analyzer. Measured results between the in-line output port (port 1) and the two input ports (port 2 and port 3) are plotted in Figure 7. As shown, return loss *S*_11_ is less than −11.3 dB over X-band and reveals a minimum value −19.6 dB at about 9.6 GHz. Insertion loss *S*_31_ is around −4.3 dB, which includes the intrinsic loss of the power combiner and the cantilever beam. In addition, the isolation *S*_23_ between the two input ports for the test signal and the reference signal is revealed to vary from −9.3 to −15.1 dB within the band of interest.

### 3.2. Phase Detection

The phase-detection capability of the proposed structure is tested with a probe station, signal generator, power splitter, phase shifter, low noise amplifier, and voltmeter. The signal provided by the signal generator is divided into two paths by the power splitter. The amplifier is utilized to amplify the signal for enough magnitude. Phase shift is introduced between the two paths by the phase shifter. According to the above analysis, once the two signals are injected into the phase detector, a DC voltage would be generated by the thermoelectric sensor. Consequently, the phase shift can be deduced from the voltage detected by the voltmeter.

**Figure 8:**
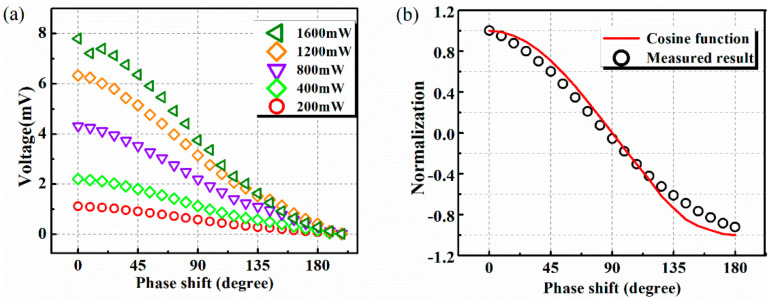
(**a**) Measured output voltages versus phase shift for different input power; (**b**) comparison between normalized measured results and theoretical cosine function.

It should be noticed that the power injected into each input port should be smaller than half of the maximum power that the beam can handle. Figure 8a shows the measured output voltage versus phase difference at 10 GHz. The magnitude of the injected power at each input port varies from 200 mW (23 dBm) to 1600 mW (32 dBm), while the phase shift, which is controlled by the phase shifter, ranges from 0 to 180°. As depicted, each plot reveals a cosinoidal relationship between the output DC voltage and the phase shift of the two input signals, which is in accordance with the theoretical analysis. Figure 8b gives the normalization of the measured result at 1200 mW, 10 GHz together with the theoretical cosine function. As can be seen, the two plots match well and show little discrepancy with each other.

**Figure 9:**
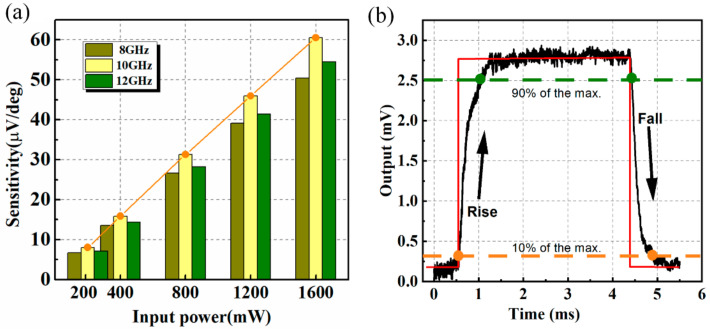
(**a**) Phase-detection sensitivities at 8, 10, and 12 GHz for different input power; (**b**) measured response time of the phase detector.

A linear relationship can be found for phase shift from 45 to 135°, which is generally used to characterize the detection sensitivity. The phase-detection sensitivity of 8, 10, and 12 GHz are displayed in Figure 9a. The sensitivities are all better than 8.04, 15.30, 28.18, 41.39, and 54.49 μV/deg at 200, 400, 800, 1200, and 1600 mW input power at 10 GHz, respectively. More importantly, the slope of the plot keeps stable which guarantees the linearity between the phase detection sensitivity and the input power. A slight decrease in sensitivity can be observed for a fixed input power when the frequency deviates from 10 GHz, and this can be attributed to the microwave performance becoming worse at the corresponding frequencies. Furthermore, the response time of the thermoelectric output during phase detection is measured. The response time is typically calculated by the average of the rise time and the fall time, which is defined by the time interval it takes between 10% and 90% of the maximum output. For the input of 500 mW, the response time is about 433 μs as presented in Figure 9b. As the response time relies on the time when thermal equilibrium is reached, it takes longer than electric signal transmission in other circuit technologies.

**Figure 10:**
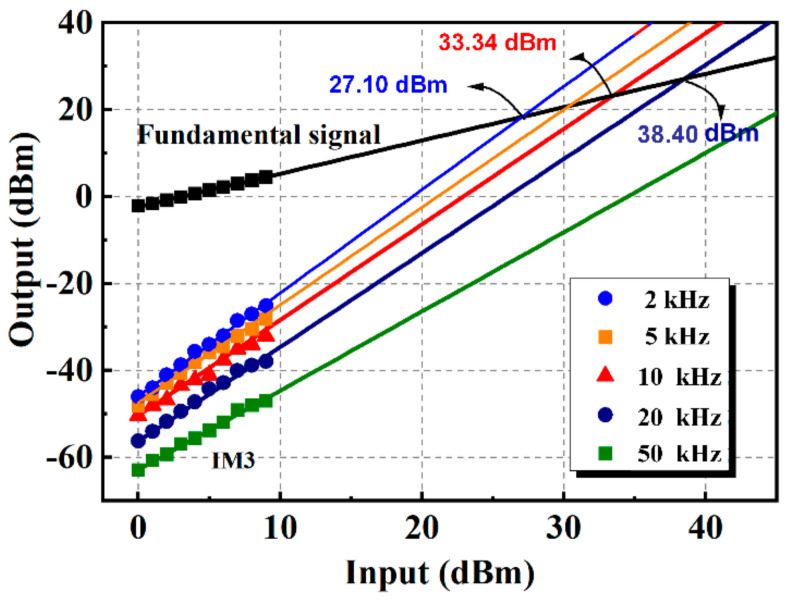
IM3 product versus frequency interval at different input power.

Based on the above measurements, the proposed phase detector has been proven to have larger power capacity and perfect linearity property for power levels up to 1600 mW when the frequencies of the two inputs are equal. However, in certain application scenarios, input signals are characterized by different frequencies, *f*_1_ and *f*_2_. In this case, the two signals modulate with each other and the in-line output signal would include several other sidebands except the fundamental frequency. Third-order intermodulation distortion (IM3) products, characterized by frequencies (2*f*_1_ − *f*_2_) and (2*f*_2_ − *f*_1_), are the ones close to fundamental frequency and influence the output frequency spectrum most [22,23,24]. Therefore, the IM3 property of the presented structure is also verified through measurement in this paper. Two signal generators are used in the experiment to produce signals with frequency interval Δ*f* and injected into the phase detector. After that, the output signal in the in-line output port is delivered to a spectrum analyzer. The magnitude of the IM3 components and the fundamental frequency input are recorded in Figure 10. The IM3 components show an increase with the input power, but its magnitude is negligible for small input signals. The third-order intercept point (IIP3), where the magnitude of the IM3 product equals the fundamental signal, can be obtained graphically by plotting the output power versus the input power, as depicted in Figure 10. A Δ*f* of 10 kHz derives an IIP3 of 33.34 dBm and a Δ*f* of 20 kHz derives an IIP3 of 38.40 dBm. It can be revealed from the measurement that the interferers generated by the presented phase detector are quite small. Therefore, it is able to handle an interference-rich environment with enough linearity.

Table 2 presents the comparison between this work and other published results in the literature. Ref. [5] is about a phase detector based on a graphene transistor, and Ref. [7] is about a phase detector based on a multiplier and fabricated with printed circuit board. Refs. [11,12] are RF MEMS phase detectors. Compared with other technologies, one of the advantages of MEMS-based phase detectors is that no power supply is needed. This will be a benefit for low power consumption applications. The power capacity and linearity region are less discussed in multipliers and other CMOS-based phase detectors, for they are usually applicable in low power situations. Thus, we didn’t find relevant data in the mentioned reference. In the aspect of phase-detection range, a common approach to realize −180~180° non-ambiguous phase detection is by using two or more channels to provide extra phase difference between different channels, as provided in Refs. [7,11], which would lead to a large occupied area. It should also be mentioned that, for the aim of improved power capacity and linearity range, the presented phase detector only couples a small portion of input power for phase detection, thus it is reasonable that the sensitivity is smaller than other detectors. The typical response time of a MEMS device with thermoelectric power sensors is several hundreds of a microsecond. In general, compared with other published results, the proposed phase detector in this work declares improved power handling ability and linearity range, small occupied size, and acceptable sensitivity.

## 4. Conclusions

In summary, a hybrid-type RF MEMS phase detector is presented in this paper. Both the in-line detection process and DC voltage output are reserved by the hybrid of a MEMS beam and a thermoelectric sensor cascaded after a power combiner. During the phase evaluation process, only fractional power is coupled by the beam and fed to the thermoelectric sensor, while the rest can transmit to subsequent circuits for reuse. Therefore, the structure can sustain much higher input power, avoid burning out, and maintain its linear behavior. Measured results show that return loss is smaller than −11.3 dB over X-band. Besides, the proposed structure is capable of handling a phase shift of 0–180° for 200–1600 mW input power level with perfect phase-detection linearity. Moreover, low intermodulation is also verified. Improved power handling ability, enhanced linearity property, and high-level integration capability are demonstrated by the presented structure. The potential application of this design lies in medium power signal microwave systems.

## Figures and Tables

**Table 1:** Structure parameters of the proposed phase detector.

Element Name and Character	Value and Unit
Dimensions of the CPW (*G*/*W*/*G*)	58 μm/100 μm/58 μm
Dimensions of the ACPS (*G*_1_/*W*_1_/*S*)	70 μm/40 μm/90 μm
Width of the beam (*w*_b_)	100 μm
Initial height of the beam (*g*_0_)	1.6 μm
Capacitance of *C*_1_, *C*_2_ and *C*_3_	0.16 pF, 0.16 pF and 0.32 pF
Resistance of *R*, *R*_1_ and *R*_2_	100 Ω, 100 Ω and 100 Ω
Length of thermopiles	150 μm
Number of the thermocouples	10 pairs
Total area of the structure	2556 μm × 960 μm

**Table 2:** Comparison between this work and other published results.

Ref.	Freq. (GHz)	Phase Range (deg)	Max. Power	Linearity Range	Sensitivity	DC Supply (V)	Size (mm^2^)	Response Time (μs)
[5]	10^−4^	−90–90	N/A	N/A	0.12 mV/deg	1.8	N/A	N/A
[7]	2.6–6	−180–180	−3 dBm	N/A	0.14 mV/deg (−5 dBm)	4	N/A	N/A
[11]	8–12	−180–180	36 dBm	21–26 dBm	36 μV/deg (27 dBm)	0	26.2	404
[12]	6–12	0–180	30 dBm	N/A	83.7 μV/deg (27 dBm)	0	>3.7	460
This work	8–12	0–180	32 dBm	23–32 dBm	21.7 μV/deg (27 dBm)	0	2.45	433

## Data Availability

The data presented in this study are available on request from the corresponding author.
